# Crosstalk along the gut-liver axis modulates glutathione and cadmium-induced hepatotoxicity

**DOI:** 10.3389/fimmu.2026.1758379

**Published:** 2026-04-28

**Authors:** Azhagu Madhavan Sivalingam

**Affiliations:** Natural Products & Nanobiotechnology Research Lab, Saveetha Institute of Basic Medical Sciences (SIBMS), Saveetha Institute of Medical and Technical Sciences (SIMATS), Saveetha University, Chennai, Tamil Nadu, India

**Keywords:** hepatoprotection, hepatotoxicity, morin, nano-formulations, oxidative stress, synergistic therapy

## Abstract

The natural flavonoid morin demonstrates promise as a dual-function agent against hepatocellular carcinoma (HCC) and chronic liver disease. Preclinical studies indicate it counteracts toxin-induced liver injury by modulating oxidative stress and inflammation via pathways like Nrf2/HO-1 and NF-κB. In HCC, morin enhances the efficacy of cisplatin by suppressing PARP1-mediated protective autophagy and DNA repair, while concurrently mitigating the drug’s nephrotoxic side effects. This capacity to simultaneously combat tumor progression and protect normal tissues positions morin as a versatile therapeutic candidate. Its clinical translation will require formal validation in human studies and the development of advanced delivery systems to overcome restrictions in bioavailability.

## 1 Introduction

**Figure 1:**
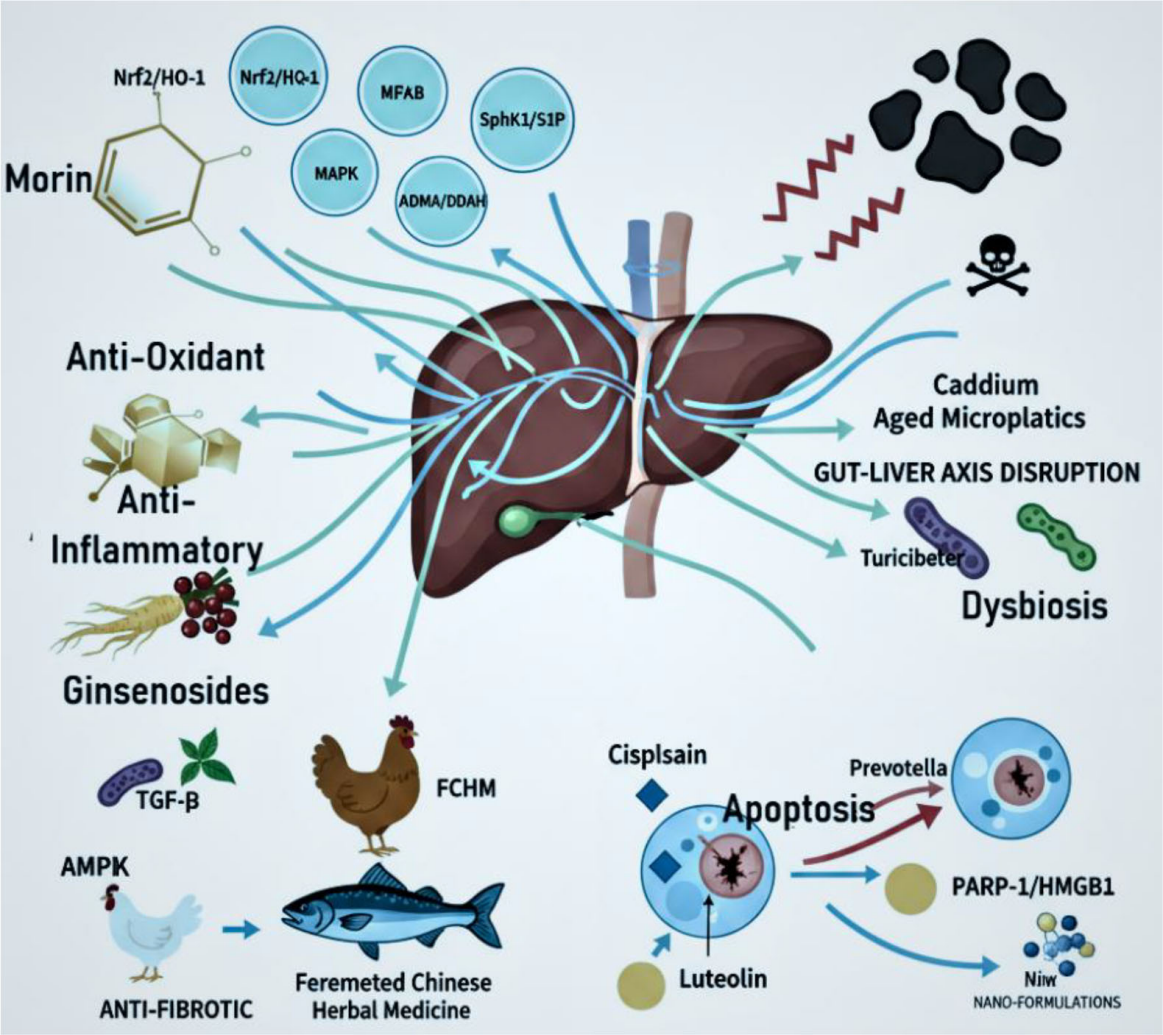
The gut–liver axis modulation in cadmium-induced hepatotoxicity. The diagram illustrates dysbiosis and microplastic-associated cadmium exposure disrupting liver homeostasis, while morin and related bioactive compounds (e.g., ginsenosides, luteolin) exert antioxidant, anti-inflammatory, antifibrotic, and pro-apoptotic effects via pathways such as Nrf2/HO-1, NF-κB, MAPK, and PARP-1. https://www.biorender.com/.

The natural flavonoid morin demonstrates potent hepatoprotective properties. It mitigates drug- and toxin-induced liver injury by suppressing oxidative stress and inflammation, largely through the modulation of Nrf2 and NF-κB signaling pathways. This multi-targeted action highlights morin’s potential as a promising therapeutic agent (1). Ginsenosides, the active compounds in ginseng, demonstrate significant hepatoprotective effects. They reduce hepatic fat accumulation, inflammation, and fibrosis by modulating key signaling pathways such as AMPK and NF-κB. These multi-targeted actions and substantial therapeutic evidence establish ginsenosides as promising candidates for the treatment of liver diseases (2, 3). Chronic low-dose cadmium exposure induces gut microbiota dysbiosis and disrupts metabolic pathways, leading to liver inflammation, steatosis, and elevated liver enzymes. Shifts in microbial populations such as increased *Prevotella* and decreased *Turicibacter* along with disturbances in bile acid metabolism and lipid homeostasis, collectively highlight the gut–liver axis as a central mechanism in cadmium-induced hepatotoxicity (4). Cisplatin induces ROS-mediated apoptosis in liver cancer cells but simultaneously triggers protective autophagy. Co-treatment with morin hydrate suppresses this survival pathway by inhibiting the PARP-1/HMGB1/Beclin1 axis, enhancing apoptotic cell death and supporting morin-cisplatin combination therapy for liver cancer (5). Luteolin protects poultry from cadmium-induced toxicity by preserving liver and intestinal function. It mitigates oxidative stress, suppresses inflammation, and helps restore gut barrier integrity. These findings identify luteolin as a promising natural agent for improving poultry health (6). Fermented Chinese herbal medicine (FCHM) alleviates cadmium toxicity by activating the Nrf2/Keap1 signaling pathway. This activation reduces oxidative stress and inflammation, while also restoring gut barrier integrity and stabilizing the gut microbiota. These results support FCHM as a promising therapeutic strategy against heavy metal–induced toxicity (7). Aged polystyrene microplastics (APS) adsorb cadmium, exacerbating hepatic injury, oxidative stress, and lipid accumulation in zebrafish. This co-exposure also causes more severe damage to intestinal barrier integrity and induces greater microbial dysbiosis. These synergistic effects along the gut–liver axis underscore the heightened ecological risk of APS–cadmium co-contamination (8). Chronic cadmium exposure disrupts gut microbiota and elevates T-β-MCA, which inhibits intestinal (Farnesoid X receptor) FXR/FGF15 signaling. This disruption triggers excessive hepatic bile acid synthesis, leading to liver injury (**Figure 1**). The mechanism is confirmed as FXR agonists reverse these effects, highlighting the gut-liver axis’s critical role (9). The APOE ϵ4 allele exacerbates cadmium-induced toxicity in male mice. Compared to ApoE3 knock-in controls, ApoE4-KI mice exposed to cadmium display more severe gut microbiota dysbiosis and greater hepatic dysfunction.

These effects included the disruption of microbial metabolic pathways, impaired host energy metabolism, and the upregulation of hepatic inflammatory and xenobiotic-processing pathways (10). Chronic cadmium exposure disrupts purine metabolism and the gut microbiota in mice, resulting in elevated uric acid levels. In bass, it induces oxidative stress and intestinal injury via the gut–liver axis. These findings reveal conserved disruptions to metabolic and immune pathways as a fundamental mechanism of cadmium toxicity (11, 12). Natural compounds like morin and ginsenosides confer hepatoprotection by modulating oxidative stress and inflammation, effectively countering cadmium-induced toxicity and supporting gut–liver axis function. However, co-exposure to environmental pollutants such as microplastics can significantly amplify this toxic damage.

>This review work aim of the hepatoprotective and therapeutic potential of natural plant-derived compounds particularly morin, ginsenosides, and key flavonoids in mitigating liver injury and disease. It focuses on their molecular mechanisms for regulating oxidative stress, inflammation, fibrosis, apoptosis, and gut–liver axis function. The scope encompasses cadmium-induced hepatotoxicity, the exacerbating effects of co-exposures such as microplastics, and the protective roles of agents including luteolin and fermented Chinese herbal medicine (FCHM). Additionally, the review highlights morin hydrate as a promising adjuvant that enhances cisplatin efficacy in hepatocellular carcinoma while reducing its systemic toxicity. By integrating current evidence on phytochemicals, heavy metal toxicity, and emerging therapeutic strategies, this work aims to support the development of multi-targeted interventions for liver diseases.

## 2 Natural bioactive as multitarget therapeutics in metabolic and liver disorders

**Figure 2:**
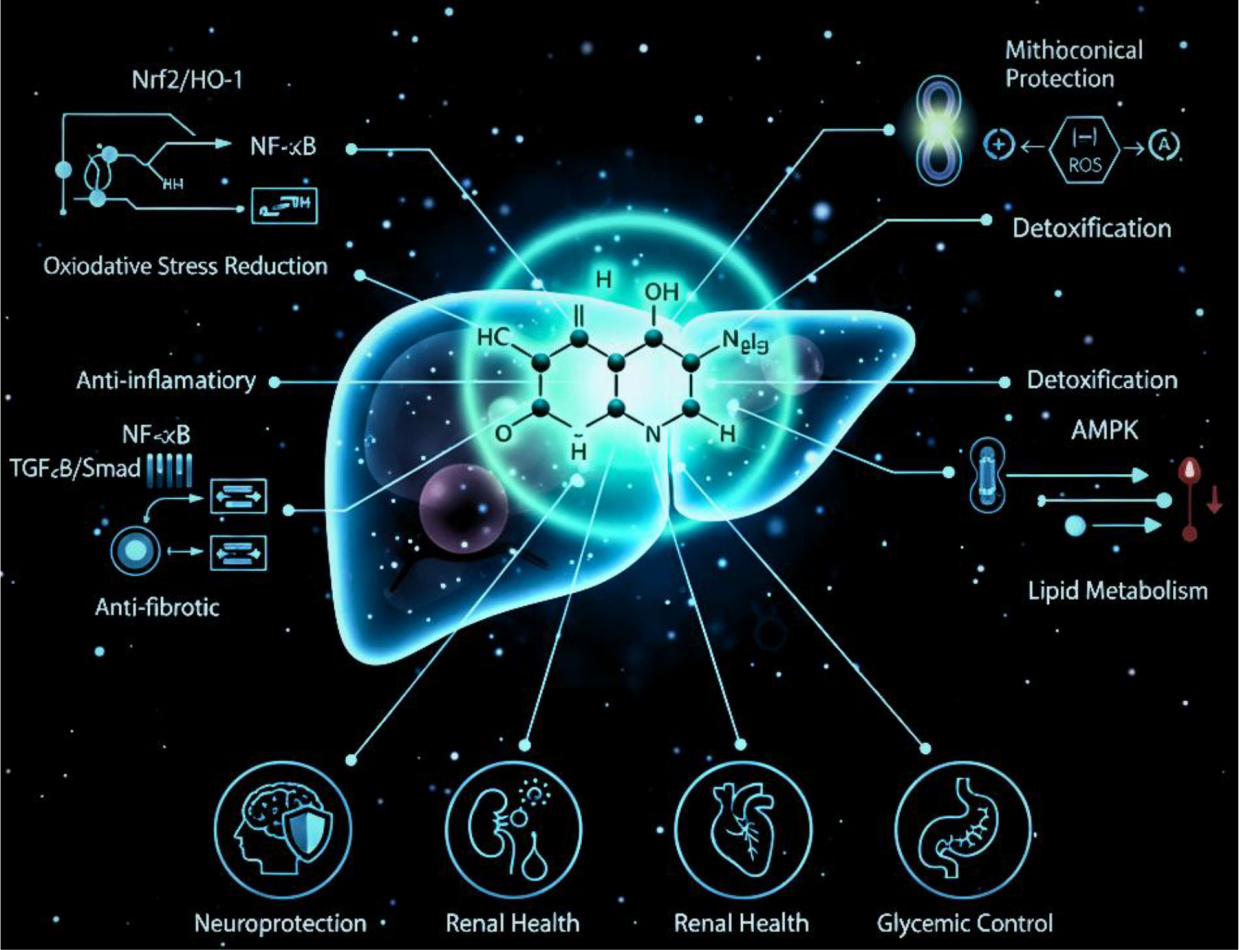
Morin modulates Nrf2/HO-1, NF-κB, TGF-β/Smad, and AMPK signaling to reduce oxidative stress, inflammation, fibrosis, and metabolic dysfunction in the liver. Its systemic benefits extend to neuroprotection, renal support, cardiometabolic health, and glucose regulation. https://www.biorender.com/.

Naturally occurring flavonoids help manage type 2 diabetes by modulating key glucose-metabolizing enzymes. The formation of metal-flavonoid complexes significantly enhances their bioavailability, positioning these compounds as promising candidates for next-generation therapeutics (13). Liver fibrosis, a consequence of chronic liver injury, can progress to cirrhosis and cancer. Given the limited effective treatments, natural flavonoids offer a promising therapeutic strategy. They mitigate fibrosis by targeting key drivers such as oxidative stress, inflammation, and hepatic stellate cell activation (14). Neurodegenerative diseases arise from disruptions in cellular processes like oxidative stress and inflammation. The Ras/Raf/MAPK signaling pathway represents a promising therapeutic target for Alzheimer’s and Parkinson’s disease, with natural compounds modulating this cascade showing strong neuroprotective potential (15). Morin hydrate demonstrates significant prophylactic potential against calcium oxalate urolithiasis, a common kidney disorder. It functions by inhibiting oxalate synthesis and crystal formation, while its antioxidant and diuretic properties provide concurrent renal protection. These findings establish morin hydrate as a promising natural therapeutic for kidney stone management (16). Epilepsy, characterized by abnormal brain activity and neuronal degeneration, arises from diverse pathogenic factors. Flavonoids counteract neurotoxicity by reducing oxidative stress, inflammation, and neuronal hyperexcitability. Their multitarget mechanisms and favorable safety profile position flavonoids as promising adjuncts to conventional antiepileptic therapies (17). Flavonoids, a widespread class of plant polyphenols, demonstrate significant therapeutic potential for neurodegenerative disorders. Dietary consumption of these compounds is linked to substantial neuroprotective benefits, highlighting their clinical value. The flavonoid tangeretin exemplifies these protective mechanisms, underscoring the promise of flavonoid-based interventions (18). Methodological heterogeneity in food antioxidant research frequently produces inconsistent results. A systematic evaluation of assessment approaches from chemical assays to cellular models reveals their distinct applications and limitations. Integrated chromatographic-chemometric methods provide a strategic and cost-effective solution for the accurate analysis of bioactive compounds (19). Kupffer cells are central drivers of hepatic inflammation during sepsis. The *Ehretia tinifolia* extract (ETME) counteracts this response by activating the protective Nrf2/HO-1 pathway while suppressing NF-κB/MAPK-mediated inflammation. This dual regulatory action highlights ETME’s potential as a therapeutic agent for liver inflammation (20, 21). *Cordyceps sinensis* polysaccharide (CS-PS) attenuates liver fibrosis by inhibiting hepatic stellate cell activation and suppressing the TGF-β/Smad signaling pathway. It also downregulates key extracellular matrix regulators, resulting in improved liver function and histopathology. These findings establish CS-PS as a promising natural anti-fibrotic agent (22, 23). TGF-β1 impairs the antifibrotic function of fetal placental mesenchymal stem cells (MSCs) by inducing Smad3-dependent autophagy, which promotes lysosomal degradation of hepatocyte growth factor (HGF). Pharmacological inhibition of autophagy restores HGF secretion and enhances the therapeutic efficacy of these MSCs against pulmonary fibrosis (**Figure 2****).** The ongoing and completed clinical trials involving representative natural compounds (e.g., curcumin, resveratrol, and related phytochemicals), including their clinical indications, trial phases, and key outcomes. For example, curcumin has been evaluated in Phase II clinical studies in pancreatic cancer and colorectal cancer patients, where oral doses up to 8 g/day demonstrated acceptable safety but limited systemic exposure, highlighting pharmacokinetic challenges. Recent progress has been made in the clinical translation of several natural compounds, with a number of candidates entering early- and mid-stage clinical trials. For instance, compounds such as curcumin, resveratrol, and epigallocatechin gallate have been investigated in Phase I and Phase II studies for indications including cancer, metabolic disorders, and inflammatory diseases. These studies generally demonstrate favorable safety profiles, although their therapeutic efficacy is often limited by poor aqueous solubility, rapid metabolism, and low systemic bioavailability. To address these challenges, considerable effort has been devoted to the development of advanced delivery systems. In particular, nano-formulation strategies—including polymeric nanoparticles, lipid-based nanocarriers, nanoemulsions, and micellar systems—have shown promise in improving solubility, stability, and pharmacokinetic behavior. Such approaches may enhance tissue targeting and therapeutic efficacy, thereby facilitating the clinical translation of natural bioactive compounds.

Therefore, modulating autophagy represents a viable strategy to enhance the efficacy of MSC-based therapies for pulmonary fibrosis (24). Oleamide (OLA), a bioactive compound derived from *Moringa oleifera*, suppresses hepatic fibrosis by inhibiting the activation of hepatic stellate cells. It reduces key fibrotic markers, including α-SMA and collagen, by blocking the TGF-β/Smad2/3 signaling pathway. This mechanism establishes OLA as a promising anti-fibrotic therapeutic candidate (25). Plant secondary metabolites enhance mitochondrial function by reducing oxidative stress and promoting mitochondrial biogenesis. They also regulate key processes such as apoptosis and mitophagy. These multifaceted actions highlight their significant therapeutic potential for treating mitochondrial disorders (26). Mitochondrially targeted CYP2E1 exacerbates alcohol-induced liver injury by inhibiting cytochrome c oxidase (CcO) in the electron transport chain. This disruption impairs mitochondrial function and amplifies oxidative stress.

Mitochondria-targeted antioxidants or CYP2E1 inhibition prevents this toxicity, highlighting CcO dysfunction as a key pathological mechanism (27). Paracetamol (APAP) overdose causes hepatorenal injury primarily through CYP450-mediated oxidative stress. Reactive oxygen and nitrogen species (ROS/RNS) play a central role in this toxicity, supporting the development of antioxidant-based interventions to mitigate APAP-induced damage. This overview also identifies critical knowledge gaps and emerging preventive strategies (28). Acetaminophen (APAP) induces toxicity in aquatic organisms like *Daphnia magna* through its reactive metabolite NAPQI. Biomarkers such as CYP370A13 expression, glutathione depletion, and oxidative stress serve as sensitive indicators of APAP exposure. These markers enhance the accuracy of environmental risk assessments for APAP contamination (29). The Jieduan-Niwan (JDNW) formula mitigates acute-on-chronic liver failure (ACLF) by suppressing E2F1-mediated hepatocyte apoptosis. It modulates both p53-dependent and p53-independent apoptotic pathways while enhancing anti-apoptotic signaling. These findings identify E2F1-related pathways as promising therapeutic targets for ACLF (30). Taraxasterol (TAR) alleviates hepatic injury by suppressing inflammation, oxidative stress, and apoptosis. It modulates the JAK/STAT and TNF signaling pathways, downregulating pro-apoptotic proteins like Bax while upregulating anti-apoptotic factors such as Bcl-2. These mechanisms establish TAR as a promising therapeutic candidate for liver diseases (31). β-Patchoulene (β-PAE) inhibits the progression of non-alcoholic fatty liver disease (NAFLD) by activating AMPK, which suppresses hepatic lipid synthesis and promotes mitochondrial fatty acid oxidation. In high-fat diet–fed rodents and fatty acid–exposed hepatocytes, β-PAE reduces weight gain, hepatocyte injury, and steatosis (**Table 1**). Notably, AMPK inhibition abolishes these benefits, confirming that β-PAE’s protective effects are AMPK-dependent (50). Oxyresveratrol alleviates hepatic steatosis by activating AMPK and inhibiting SREBP-1c–mediated lipogenesis, demonstrating anti-NAFLD efficacy comparable to atorvastatin in animal models (51). Catalpol reduces hepatic steatosis by activating AMPK to suppress lipogenesis and promote fatty acid oxidation, thereby lowering lipid accumulation in NAFLD models (52).

**Table 1:** Multi-targeted mechanisms of hepatoprotection.

Aspect category	Specific mechanism	Molecular target	Biological effect	Experimental model	Outcome	Signaling pathway	Oxidative stress interaction	Inflammation interaction	Apoptosis interaction	Clinical relevance
Antioxidant Activity	Free radical scavenging	ROS/RNS	Reduces lipid peroxidation	CCl_4_-induced rat hepatotoxicity	↓ MDA levels; ↑ GSH reserves	Nrf2/ARE	Direct neutralization	Indirect suppression	Anti-apoptotic	Prevention of oxidative liver damage (32).
Anti-inflammatory	NF-κB inhibition	IκB kinase complex	Suppresses TNF-α, IL-6, IL-1β	LPS-stimulated Kupffer cells	↓ Pro-inflammatory cytokines	NF-κB	↓ ROS-induced activation	Direct inhibition	↓ Caspase-3 activation	Alleviates hepatitis (33).
Antifibrotic	TGF-β1/Smad suppression	TGF-β receptor I	Inhibits HSC activation	Thioacetamide-induced fibrosis	↓ Collagen deposition; ↓ α-SMA	TGF-β/Smad	↓ Oxidative drivers	↓ Inflammatory triggers	↓ HSC apoptosis	Slows cirrhosis progression (25, 34).
Mitochondrial Protection	Complex I/III stabilization	Mitochondrial electron transport	Maintains ATP synthesis; ↓ Cyt c release	Ethanol-induced injury	↑ Mitochondrial membrane potential	SIRT1/PGC-1α	↓ mtROS	Preserves integrity	↓ Intrinsic apoptosis	Mitigates alcoholic liver disease (35).
Detoxification	CYP2E1 downregulation	Cytochrome P450 enzymes	Reduces toxic metabolite generation	Acetaminophen overdose model	↓ NAPQI formation; ↑ GST activity	Nrf2	↓ CYP2E1-derived ROS	Prevents secondary inflammation	↓ Necrosis	Antidote adjunct potential (36).
Antiapoptotic	Caspase cascade inhibition	Caspase-9/3	Blocks mitochondrial apoptosis pathway	D-GalN/LPS-induced fulminant hepatitis	↓ DNA fragmentation; ↑ Bcl-2/Bax ratio	PI3K/Akt	Counters ROS-mediated apoptosis	↓ Inflammation-induced death	Direct inhibition	Reduces hepatocyte loss (31).
Lipid Metabolism	AMPK activation	AMP-activated protein kinase	Enhances β-oxidation; ↓ Lipogenesis	NAFLD mouse model	↓ Hepatic triglycerides; ↑ Fatty acid oxidation	AMPK/SREBP-1c	↓ Oxidative lipotoxicity	↓ Steatohepatitis	↓ Lipotoxicity apoptosis	NAFLD/NASH therapy target (37).
Iron Chelation	Fe²^+^/Fe³^+^ binding	Labile iron pool	Prevents Fenton reactions	Iron-overload model	↓ LIP; ↓ Ferritin accumulation	Nrf2/Hepcidin	Direct ROS prevention	↓ Iron-induced inflammation	↓ Ferroptosis	Hemochromatosis management (38).
Gut-Liver Axis	Intestinal barrier restoration	TLR4/MyD88	Reduces endotoxin translocation	Alcoholic liver disease model	↓ Serum endotoxin; ↓ Bacterial translocation	TLR4/NF-κB	↓ ROS-driven barrier damage	↓ Gut inflammation	Preserves enterocytes	Addresses leaky gut comorbidity (39).
Immunomodulation	Treg cell promotion	Foxp3 transcription	Shifts Th1/Th2 balance	Autoimmune hepatitis model	↑ Tregs; ↓ IFN-γ/IL-17	STAT3/TGF-β	↓ Immuno-oxidative stress	Direct cytokine balance	↓ Autoimmune apoptosis	Autoimmune hepatitis potential (40).
Angiogenesis	VEGF inhibition	VEGF receptor 2	Suppresses pathological vasculature	Portal hypertension model	↓ Sinusoidal remodeling; ↓ Portal pressure	VEGFR2/PI3K-Akt	↓ ROS-driven angiogenesis	↓ Inflammation-linked VEGF	↓ Hypoxic apoptosis	Prevents portal hypertension (41).
DNA Protection	PARP-1 inhibition	Poly(ADP-ribose) polymerase	Reduces NAD^+^ depletion	Oxidative DNA damage model	↓ PARylation; ↑ DNA repair	PARP-1/P53	Prevents ROS-induced DNA breaks	↓ Inflammation-induced damage	↓ PARP-mediated apoptosis	Limits genotoxic injury (42).
Bile Acid Homeostasis	FXR activation	Farnesoid X receptor	Enhances bile acid export	Cholestasis model	↓ Serum bile acids; ↑ BSEP expression	FXR/SHP	↓ Bile acid-induced ROS	↓ Cholestatic inflammation	↓ Bile acid apoptosis	Cholestasis treatment (43).
Autophagy	mTORC1 inhibition	mTOR complex 1	Induces protective autophagy	Steatosis-hepatocarcinoma model	↑ LC3-II; ↓ p62 aggregation	AMPK/mTOR	Clears ROS-damaged organelles	Removes inflammasomes	↓ Apoptotic burden	Prevents steatosis-to-cancer shift (44).
Antiviral	NS3 protease inhibition	HCV NS3/4A protease	Inhibits viral replication	HCV replicon system	↓ Viral RNA; ↑ Interferon response	JAK/STAT	↓ Virus-induced oxidative burst	↓ Viral inflammation	↓ Infection-related apoptosis	Adjunct in viral hepatitis (45).
Metal Homeostasis	Cu²^+^/Zn²^+^ regulation	Metallothionein expression	Prevents copper-induced injury	Copper-loaded hepatocytes	↑ Metallothioneins; ↓ Copper toxicity	MTF-1	Chelates redox-active metals	↓ Metal-induced inflammation	↓ Copper-mediated apoptosis	Wilson’s disease application (46).
Glycemic Control	IRS-1 phosphorylation enhancement	Insulin receptor substrate 1	Improves insulin sensitivity	Diabetic rat model	↓ Fasting glucose; ↑ Glycogen synthesis	IRS-1/PI3K-Akt	↓ Glycoxidative stress	↓ Diabetes-related inflammation	↓ Glucolipotoxicity	Addresses diabetic hepatopathy (47).
Epigenetic Regulation	HDAC inhibition	Histone deacetylase 3	Reactivates silenced antioxidant genes	Aging liver model	↑ SOD2 expression; ↓ Senescence markers	HDAC3/Nrf2	Restores redox gene expression	↓ Senescence-associated inflammation	↓ Senescence apoptosis	Anti-aging hepatoprotection (48).
Synergistic Effects	Pharmacokinetic optimization	Drug transporters (OATP1B1)	Enhances bioavailability of co-therapies	Silymarin + Morin co-administration	↑ Therapeutic efficacy; ↓ Side effects	Multi-target potentiation	Augments antioxidant defenses	Broad anti-inflammatory	Enhanced anti-apoptotic	Foundation for combination therapies (49).

This table high quality and information clarity and direct relevance to the scope of the current manuscript.

“↓” indicates downregulation (decrease). “↑” indicates upregulation (increase).

Lipopolysaccharide (LPS) crosses the compromised intestinal barrier and triggers hepatic inflammation via the gut-liver axis. It activates pro-inflammatory responses in Kupffer cells and other liver cells, exacerbating liver injury. Maintaining intestinal barrier integrity is therefore essential to prevent LPS translocation and limit disease progression (39, 53). Curcumin ameliorates hepatic steatosis by improving intestinal barrier integrity and decreasing lipopolysaccharide (LPS)-mediated hepatic inflammation. It suppresses activation of the TLR4/NF-κB signaling pathway and downregulates proinflammatory cytokines. These effects contribute to reduced hepatic lipid accumulation and inflammatory progression. Collectively, curcumin demonstrates promising nutraceutical potential for the management of non-alcoholic fatty liver disease (NAFLD) (54). Sulforaphane (SFN) mitigates non-alcoholic fatty liver disease (NAFLD) by modulating the gut–liver axis. It restores gut microbiota balance, strengthens intestinal barrier integrity, and suppresses LPS-induced hepatic inflammation. These multi-target actions position SFN as a promising nutraceutical candidate for NAFLD therapy (55). Regulatory T cells (Tregs) present significant therapeutic potential for autoimmune liver diseases and transplantation tolerance. They provide targeted immunomodulation with fewer adverse effects than chronic immunosuppression. Recent advances in Treg-based therapies highlight their promising translational prospects for clinical hepatology (56). Autoimmune hepatitis (AIH) is a progressive liver condition. Its development is linked to an imbalance in gut microbiota, which triggers the activation of harmful T cells. This connection emphasizes the critical role of the gut-liver-immune axis in the disease process and identifies it as a valuable focus for novel treatments (57). The Hepatitis C virus (HCV) relies on the host’s production of phosphatidylcholine for its own replication, a mechanism driven by the viral NS3/4A protease. New inhibitors that target this protease successfully block viral replication, offering a dual therapeutic strategy: they inhibit the viral enzyme and disrupt the host lipid environment essential for the virus (45, 58). Tumor cells are especially vulnerable to ROS-based therapies due to their limited antioxidant defenses. Copper-doped carbon dots (Cu-CDs) exploit this weakness by selectively targeting metallothionein-2 (MT-2), a key antioxidant protein. This ApoE-dependent interaction triggers severe oxidative stress and inhibits tumor growth (59). The global impact of diabetes is closely connected to the development of early insulin resistance, which stems from impaired signaling at the insulin receptor (INSR). While multiple treatments are available, very few directly address the central INSR/IRS pathway. This underscores the necessity for therapies that can more precisely regulate this key signaling mechanism to achieve better diabetes management (60). Globally, liver diseases are responsible for nearly two million deaths per year, and oxidative stress is a key contributing factor. N-acetylcysteine (NAC) protects the liver via diverse antioxidant and immunomodulatory actions. Given its dose-dependent effectiveness and safety, NAC is a strong candidate for repurposing as a clinical treatment for liver disease (61). Phytochemicals in common plant-based beverages can alter drug metabolism by modulating enzymes and transporters like CYP450 and OATP1B1. This risk is heightened by genetic variants such as the OATP1B1 R57Q polymorphism, which impairs drug transport and can lead to systemic toxicity (62, 63). The natural flavonoid morin provides broad hepatoprotection by regulating oxidative stress, inflammation, and fibrosis through pathways like Nrf2 and NF-κB. Its multi-target mechanisms also indicate therapeutic potential for metabolic, renal, and neurodegenerative disorders.

## 3 Natural bioactive compounds advancing emerging therapeutics across chronic diseases

**Figure 3:**
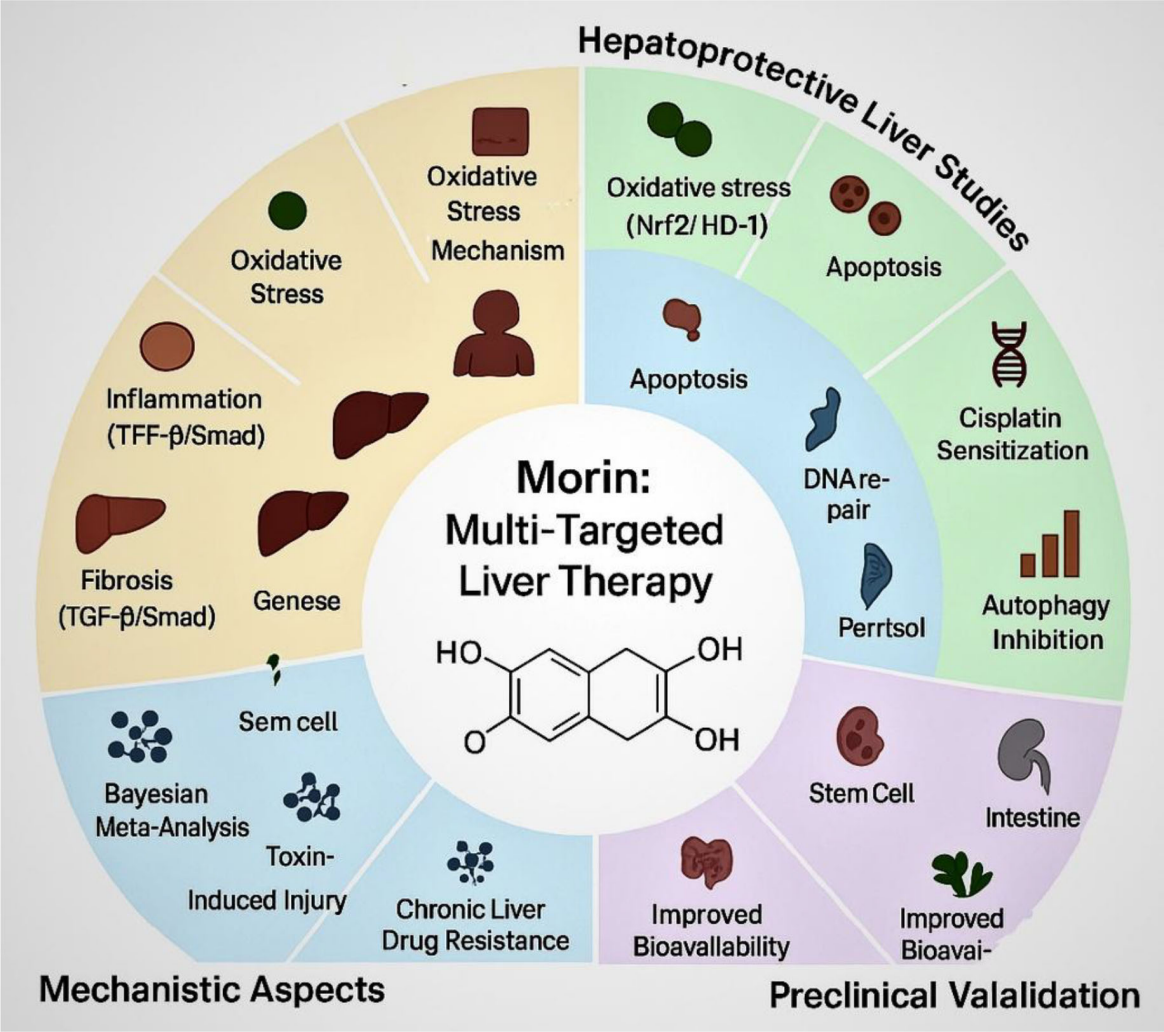
Morin acts through diverse molecular pathways including Nrf2/HD-1, TGF-β/Smad, apoptosis, DNA repair, and autophagy inhibition. These combined mechanisms underpin its hepatoprotective, anti-fibrotic, and chemosensitizing effects. Preclinical studies validate morin as a promising multi-targeted agent for liver disease treatment. https://www.biorender.com/.

Medicinal plants contain bioactive compounds like triterpenoids and curcumin, which possess significant therapeutic potential against major diseases. Their mechanisms of action include activating GLUT4 and inhibiting NF-κB. For broader clinical use, however, these benefits require systematic and well-designed clinical validation (64). Plant-derived bioactive compounds exhibit strong therapeutic potential owing to their antioxidant, anti-inflammatory, and antimicrobial properties. Their wide-ranging applications in disease prevention and treatment underscore their medical significance (65). Phytoconstituents demonstrate therapeutic potential for breast cancer by inducing apoptosis, inhibiting proliferation, and preventing metastasis. Many of these plant-based compounds also exhibit anti-angiogenic properties and can enhance the efficacy of conventional therapies (66). Plant-derived bioactive compounds and essential oils show broad therapeutic potential for treating infections, inflammation, and chronic diseases. Their bioavailability and clinical application are advanced through improved extraction techniques and nanoformulations, underscoring their promise for integrative medicine (67). Medicinal plants provide powerful antimicrobials that fight multidrug-resistant bacteria. These compounds often enhance the effect of standard antibiotics and have promising applications in pharmaceuticals, food preservation, and cosmetics (68). *Peucedanum ostruthium* shows anti-tubercular potential in silico, with nine bioactive compounds mapped to 256 TB-related genes. Kaempferol-3-O-rutinoside exhibits strong binding to the essential MmpL3 transporter, supporting its further experimental development as a therapeutic applicant (69). Plant- and marine-derived bioactive compounds, including alkaloids, terpenes, and phenolics, show strong anticancer activity against prostate cancer. Their therapeutic potential reinforces the continued value of natural products in developing new oncology treatments (70). Phytochemicals can improve diabetic wound healing by regulating oxidative stress and inflammation, with agents like resveratrol also supporting glycemic control and reducing complications. Integrating these plant-based therapies with emerging technologies may further advance diabetes management (71, 72). Liver diseases cause over two million deaths each year, underscoring the need for new hepatoprotective agents. Computational analyses identified 5′-hydroxymorin as a promising lead with strong binding affinity and favorable drug-like features, offering an efficient route for developing targeted liver therapies (73). Phytochemical antioxidants from plants and marine algae provide multitarget benefits, including free radical scavenging and anti-inflammatory effects. Their broad protective actions highlight strong nutraceutical potential for disease prevention and treatment (74). Plant-derived phytochemicals show strong chemopreventive activity against breast cancer by selectively modulating important signaling pathways and offering a safer profile than many conventional treatments (**Figure 3****).** These features make them promising complementary or alternative options in cancer management (75). UV radiation increases oxidative stress and skin cancer risk, but medicinal plants especially those used in Traditional Chinese Medicine offer photoprotection by modulating key cellular pathways. Modern delivery systems have further improved their stability and bioavailability, strengthening their dermatological potential (76).

Bioactive compounds from medicinal plants offer promising solutions to the growing AMR crisis by disrupting microbial proteins and modulating host immune responses. Their dual mechanisms underscore strong translational potential for both human and veterinary medicine (77). Liriodendrin, a lignan from *Boerhaavia diffusa*, demonstrates strong antioxidant, anti-inflammatory, and cardioprotective effects. It shows therapeutic potential in models of myocardial infarction, liver injury, and even SARS-CoV-2 infection. These broad actions position liriodendrin as a promising multifaceted therapeutic (78). Phytoconstituents like flavonoids and alkaloids demonstrate broad pharmacological effects, including antioxidant, anti-inflammatory, and anticancer activities. Their capacity to modulate multiple molecular targets offers significant therapeutic potential for managing chronic and infectious diseases. While advances in bioinformatics and nanoformulations are enhancing their pharmacokinetic profiles and clinical applicability, challenges such as standardization, dosage optimization, and translational validation hinder their widespread clinical adoption.

## 4 Bioactive ginsenosides in metabolic and inflammatory liver disorders

Non-alcoholic fatty liver disease (NAFLD) can progress to a more severe stage called NASH, which involves inflammation and liver scarring. Ginseng and its active compounds, ginsenosides, counteract this progression through anti-adipogenic, anti-inflammatory, antioxidant, and anti-scarring mechanisms. These consistent experimental results affirm ginseng’s potential as a treatment for steatosis-driven liver conditions (79). Ginsenoside Rg3 demonstrates therapeutic benefits for various liver conditions, including NAFLD and liver cancer. It acts by modulating apoptosis, autophagy, oxidative stress, and inflammatory responses through key signaling pathways. Supported by progress in targeted delivery systems, these multifaceted mechanisms underscore its significant potential for clinical application (80). Non-alcoholic fatty liver disease (NAFLD) is rising globally and still lacks effective therapeutic options. Ginseng shows promising potential by targeting multiple pathways, including lipid metabolism, inflammation, oxidative stress, and gut microbiota regulation. These integrated actions highlight its clinical relevance for NAFLD management (81). Ginsenoside Rg1, a key bioactive component of ginseng, shows significant potential for treating Non-alcoholic fatty liver disease (NAFLD), a condition with limited therapeutic options. Its efficacy is demonstrated through the reduction of liver fat, improvement of liver function markers, and regulation of specific genes such as Atf3 and Acox2, supporting its development as a targeted therapy (82). By targeting both the gut and the liver, Ginsenoside extract (GE) from *Panax ginseng* alleviates NAFLD in high-fat diet-fed mice. It improves gut microbiota composition and barrier function, thereby reducing systemic inflammation, while simultaneously rebalancing lipid metabolism genes. These combined prebiotic and metabolic effects highlight GE’s potential as a comprehensive therapeutic agent (83). Ginsenoside Rf, a compound unique to *Panax ginseng*, mitigates early-stage non-alcoholic fatty liver (NAFL) by modulating lipid metabolism, inflammation, and key signaling pathways. qPCR analysis confirmed that Rf regulates critical genes, including ANXA2, BAZ1A, DNMT3L, and MMP9, thereby clarifying its underlying mechanism of action. These findings identify Rf as a promising therapeutic candidate for NAFL (84). Ginsenoside F2 (GF2), a bioactive compound unique to *Panax ginseng*, alleviates metabolic dysfunction–associated steatotic liver disease (MASLD) by modulating liver X receptor α (LXRα). By selectively recruiting LXRα coregulators, GF2 reduces hepatic lipid accumulation and suppresses inflammation of identify GF2 as a promising therapeutic candidate for metabolic liver diseases (85–87).

Ginsenoside Rh4 alleviates non-alcoholic fatty liver disease (NAFLD) by modulating the gut–liver axis. It reduces hepatic steatosis and inflammation, restores gut microbiota balance, and activates the FXR pathway. These findings support its potential as a promising therapeutic candidate for NAFLD (88). Ginseng saponin extract and its specific ginsenosides improve NAFLD outcomes by reducing hepatic steatosis, fibrosis, and inflammation. Their mechanism involves enhancing mitophagy, suppressing NLRP3 inflammasome activation in hepatocytes, and modulating key metabolic regulators. These combined actions underscore their therapeutic potential for NAFLD (89, 90). Ginsenosides, the primary bioactive compounds in *Panax ginseng*, exert potent anti-inflammatory effects by suppressing proinflammatory cytokines and inhibiting signaling pathways such as NF-κB. Specific ginsenosides, including Rb1 and Rg3, demonstrate significant efficacy in preclinical models of inflammatory disease, supporting their clinical potential for managing a broad range of inflammatory conditions (91). *Panax ginseng* modulates the gut microbiota to enhance the production of the bioactive ginsenoside compound K (CK). To overcome CK’s poor bioavailability, an albumin-based nanoformulation (nabCK) was developed for improved hepatic targeting. In NAFLD models, nabCK significantly reduces steatosis and fibrosis by inhibiting the mTOR pathway (92, 93). *Lycium ruthenicum* Murray demonstrates protective effects against ethanol-induced alcoholic liver disease in murine models. It alleviates hepatic injury by activating the Nrf2/HO-1 antioxidant pathway and suppressing NF-κB–mediated inflammation, highlighting its potential as a therapeutic applicant for ALD (94). Ginsenoside Rg1 alleviates cholestatic liver injury by activating the SIRT1 pathway in hepatocytes. It reduces hepatic inflammation and oxidative stress by modulating the SIRT1/Nrf2/NF-κB axis, thereby restoring liver function. The absence of these protective effects in SIRT1-knockout mice confirms the mechanism’s specificity (95). Antrodan, a β-glucan from *Antrodia cinnamomea*, ameliorates NAFLD in mice by reducing serum lipids, oxidative stress, and inflammation. Its mechanism involves activating the AMPK/SIRT1 pathways and suppressing lipogenic regulators like PPARγ and SREBP-1c, highlighting its potential as a therapeutic candidate for NAFLD (96). Ginsenoside Rg3 exhibits anti-fibrotic effects in both hepatic and cardiac tissues by modulating the TGF-β/Smad signaling pathway. In cardiac tissue, it further attenuates fibrosis by restoring acylase 1 (ACY1) expression. These findings position Rg3 as a promising broad-spectrum anti-fibrotic agent (97, 98). α-Mangostin protects against drug-induced acute liver injury by mitigating oxidative stress and suppressing inflammation. It functions by restoring key antioxidant enzymes and inhibiting proinflammatory cytokine production, mediated through the inhibition of MAPK and NF-κB signaling pathways. These mechanisms underscore its therapeutic potential (99). Ginsenoside Rh2-O suppresses hepatocellular carcinoma by inducing cell cycle arrest and apoptosis via activation of both intrinsic and extrinsic pathways. In contrast, 2-Deoxy-Rh2 inhibits breast cancer through a distinct mechanism, disrupting mitochondrial metabolism and promoting autophagy. These findings position Rh2-O as a candidate for liver cancer therapy and 2-Deoxy-Rh2 for breast cancer treatment (100, 101). Hepatitis B virus (HBV) infection can progress to life-threatening liver fibrosis, cirrhosis, and hepatocellular carcinoma (HCC). Existing *in vivo* models fail to fully recapitulate HBV pathogenesis and immune responses due to the virus’s narrow human and chimpanzee tropism. This study establishes the first chemically induced cirrhosis/HCC model that mirrors the pathophysiological features of HBV infection (102). In murine models of acute liver failure, early-stage liver stem cells (LSCs) demonstrate superior hepatoprotective effects compared to mature hepatocytes. Both the YE and R5 LSC subtypes reduced liver injury markers and improved immune balance, with the less mature YE cells exhibiting the greatest efficacy (**Table 2**). These results suggest that the developmental stage of LSCs is a key factor for therapeutic advantage in acute liver failure (113). The pharmacokinetic properties including intestinal permeability, first-pass metabolism, microbiota-mediated biotransformation, plasma half-life, and systemic availability is essential for translational relevance. Clarifying compound–gut microbiota interactions and whether microbial metabolism enhances or limits therapeutic efficacy would strengthen the gut–liver axis perspective. Incorporating *in vivo* pharmacokinetic data or clinical evidence would enhance scientific rigor.

**Table 2:** Therapeutic roles of ginsenosides in liver diseases. “↓” indicates downregulation (decrease). “↑” indicates upregulation (increase).

Ginsenoside	Disease target	Primary mechanism	Model system	Observed effects	Key pathways	Anti-inflammatory	Antioxidant	Anti-fibrotic	Anti-apoptotic	Evidence level	Therapeutic potential
Rb1	Alcoholic Liver Disease	Inhibits oxidative stress & apoptosis	Rat hepatocytes	Reduces ALT/AST, ROS, and hepatic steatosis	↑ Nrf2/HO-1	✓	✓	✗	✓	Phase II trials	Hepatoprotection (103).
Rg1	NAFLD/NASH	Activates AMPK	HFD-fed mice	Decreases lipid accumulation; improves insulin sensitivity	↓ AMPK/SREBP-1c	✓	✓	✓	✓	Preclinical	Metabolic regulation (96).
Rg3	Hepatic Fibrosis	Suppresses HSC activation	CCl_4_-induced rats	Reduces collagen I/III and α-SMA expression	↓ TGF-β/Smad	✓	✓	✓	✗	Phase I/II trials	Anti-fibrotic (104).
Rd	Drug-Induced Injury	Enhances mitochondrial function	Acetaminophen-treated mice	Increases GSH; reduces CYP2E1 and necrosis	↓ JNK/ERK	✓	✓	✗	✓	Preclinical	Detoxification (105).
Rh2	Hepatocellular Carcinoma	Induces tumor apoptosis	HepG2 cells	Increases caspase-3; decreases Bcl-2 and tumor growth	↓ PI3K/Akt	✗	✗	✗	✓	*In vitro*	Anti-tumor (100, 106).
CK	Viral Hepatitis	Modulates immune response	HBV-transgenic mice	Reduces HBV DNA and inflammation; increases IFN-γ	↓ TLR4/NF-κB	✓	✓	✗	✗	Preclinical	Immunomodulation (102).
Re	Ischemia-Reperfusion Injury	Reduces ER stress	Mouse I/R model	Decreases ALT/AST, GRP78, and necrosis	↓ PERK/eIF2α	✓	✓	✗	✓	Animal studies	Ischemia protection (107).
Rf	Cholestasis	Regulates bile acid transport	ANIT-induced rats	Lowers bilirubin; upregulates BSEP	↑ FXR	✗	✓	✗	✗	Preclinical	Cholestasis management (108).
F1	NAFLD/NASH	Promotes fatty acid oxidation	Oleic acid-treated HepG2	Reduces triglycerides; upregulates PPAR-α	↑ PPAR-α/γ	✗	✓	✗	✓	*In vitro*	Lipid metabolism (109).
Rg5	Cirrhosis	Blocks TGF-β1 signaling	BDL rats	Reduces hydroxyproline and TIMPs; slows fibrosis	↓ TGF-β1/Smad2/3	✓	✗	✓	✗	Phase I trials	Fibrosis suppression (110).
Rh1	Alcoholic Liver Disease	Enhances autophagy	Ethanol-fed mice	Increases LC3-II and Beclin-1; reduces apoptosis	↓ mTOR	✓	✓	✗	✓	Preclinical	Autophagy induction (111).
Rg2	Hepatic Fibrosis	Inhibits epithelial-mesenchymal transition	TGF-β1-stimulated LX2 cells	Reduces vimentin; increases E-cadherin and inhibits HSC migration	↓ Wnt/β-catenin	✓	✗	✓	✗	*In vitro*	EMT inhibition (112).
Ro	Autoimmune Hepatitis	Suppresses T-cell activation	ConA-induced mice	Reduces IL-17 and hepatic necrosis; increases Tregs	↓ STAT3	✓	✗	✗	✗	Animal studies	Immunosuppression (113).
Ra2	Hepatocellular Carcinoma	Anti-angiogenic activity	Xenograft mice	Decreases VEGF and microvessel density; suppresses metastasis	↓ HIF-1α/VEGF	✗	✗	✗	✓	Investigational	Anti-metastatic (114, 115)
Rb2	NAFLD/NASH	Modulates gut-liver axis	HFD + probiotics in rats	Increases butyrate; decreases LPS and steatohepatitis	↓ TLR4/MyD88	✓	✓	✓	✗	Preclinical	Microbiome regulation (116).
Rg6	Drug-Induced Injury	Upregulates UGT enzymes	Diclofenac-treated rats	Enhances glucuronidation; reduces hepatotoxicity	↑ CAR/PXR	✗	✓	✗	✗	Phase I trials	Metabolic detox (117).
Rh4	Hepatic Metastasis	Inhibits matrix metalloproteinases	CRC liver metastasis model	Decreases MMP-9 and invasion; increases E-cadherin	↓ ERK/MMP-9	✗	✗	✗	✓	*In vitro*/animal	Metastasis suppression (118).

Inhibition of non-CYP enzymes especially UDP-glucuronosyltransferases (UGTs) significantly predicts high risk for drug-induced liver injury (DILI). Critically, UGT *inhibition* (not substrate activity) correlates with hepatotoxic potential.A drug’s inhibition of UGT enzymes is a direct indicator of increased hepatotoxicity risk. Integrating UGT inhibition data into the rule-of-two model significantly enhances the prediction accuracy for drug-induced liver injury (DILI) (119). Hepatocellular carcinoma progression is driven by pathological angiogenesis and an immunosuppressive microenvironment. Combining antiangiogenic agents, such as saikosaponin b2 which targets the VEGF/ERK/HIF-1α pathway, with immunotherapy enhances treatment efficacy. The identification of predictive biomarkers could further optimize these combination strategies (114, 115). Colorectal cancer frequently metastasizes to the liver, a major contributor to its high mortality. Twenty key molecular drivers involved in proteolysis and angiogenesis have been identified as potential biomarkers and therapeutic targets. Understanding these mechanisms could inform new strategies to prevent metastasis and improve patient outcomes (120). The development of colorectal cancer liver metastases depends on complex tumor-stroma interactions and a supportive metastatic niche. Key pathways such as c-MET and TGF-β, modulated by factors like MACC1, are central to this process. Emerging therapeutic targets including PRL3 and L1CAM demonstrate promising anti-metastatic potential, highlighting critical avenues for intervention (118). Ginsenosides from *Panax ginseng* combat liver diseases by modulating key pathways involved in lipid metabolism, oxidative stress, and inflammation. While their preclinical efficacy is promising, clinical translation has been limited by poor bioavailability. Recent advances in nanoformulations and targeted delivery systems are now overcoming this barrier, significantly enhancing their therapeutic potential.

## 5 Gut–liver crosstalk in cadmium-induced hepatotoxicity

Luteolin and fermented Chinese herbal medicines protect livestock from cadmium-induced damage by mitigating oxidative stress and inflammation. Both agents activate the Nrf2-Keap1 pathway, which enhances antioxidant defenses and restores gut barrier integrity. This shared mechanism supports their potential as sustainable interventions for heavy metal toxicity in animal production (6, 7). Aged polystyrene microplastics exacerbate cadmium-induced hepatotoxicity in zebrafish by amplifying oxidative stress and inflammatory responses. This co-exposure causes intestinal barrier dysfunction and gut microbiota dysbiosis, driving synergistic liver injury via the gut-liver axis. The toxicity is sphingolipid and pentose phosphate pathway metabolism, highlighting a significant ecological risk (8). Cadmium exposure induces gut dysbiosis and increases intestinal levels of T-βMCA, which inhibits the FXR/FGF15 signaling pathway. This suppression subsequently promotes excessive hepatic bile acid synthesis, resulting in inflammation and liver injury. The central role of the gut-liver axis is demonstrated by the reproducibility of these effects through fecal microbiota transplantation and their reversal upon FXR activation (9). Cadmium exposure induces gut microbiota dysbiosis, elevating intestinal T-βMCA and subsequently inhibiting hepatic FXR/FGF15 signaling. This inhibition increases bile acid synthesis, driving ductular reaction and liver injury. The pivotal role of the gut-liver axis is confirmed as these effects are reproduced through fecal microbiota transfer and mitigated by FXR activation (11, 12). Chronic cadmium exposure impairs gut health, increasing hepatic cadmium accumulation and subsequent liver injury. Melatonin counteracts this damage by restoring gut microbiota, reducing toxic metabolites, and activating intestinal FXR signaling. These findings underscore the central role of the gut-liver axis in both cadmium-induced toxicity and the protective mechanism of melatonin (121, 122). Individual exposure to microplastics or cadmium induces intestinal and liver injury in broilers via TLR4/NF-κB pathway activation. However, combined exposure resulted in unexpectedly lower toxicity than either pollutant alone. This antagonistic interaction reveals the complex, non-additive nature of combined pollutant effects (123). An engineered probiotic expressing metallothionein alleviates cadmium-induced liver injury by activating the Nrf2-Keap1 antioxidant pathway and restoring intestinal barrier integrity. Transcriptomic analysis identified key genes implicated in cadmium toxicity, providing mechanistic insight. These findings support the development of probiotic-based interventions for cadmium detoxification (124, 125). Cadmium exposure impairs growth and hepatic function in male Shaoxing ducks by inducing oxidative stress, disrupting mitochondrial ultrastructure, and activating pyroptosis and fibrogenesis. These detrimental effects are linked to suppression of the Nrf2 antioxidant pathway. Lycium barbarum polysaccharide counteracts this hepatotoxicity by preserving mitochondrial integrity and activating Nrf2 signaling, supporting its potential as a protective agent against cadmium-induced liver damage (126). Chronic low-dose cadmium exposure induces liver injury via gut microbiota dysbiosis, even without overt intestinal damage. This toxicity is characterized by specific microbial alterations and disrupted bile acid metabolism. These findings establish the gut-liver axis as a primary mediator of cadmium toxicity and a potential target for therapeutic intervention (4). Cadmium exposure induces fetal growth restriction by damaging the placental barrier and disrupting essential cellular processes. Melatonin counteracts these effects by alleviating placental injury and restoring gut microbiota balance. The role of the gut-placental axis is demonstrated by the replication of melatonin’s protective effects through fecal microbiota transplantation (127). The intervention alleviates cadmium-induced liver injury by restoring gut microbiota, specifically enriching beneficial Ruminococcaceae. This microbial rebalancing reduces hepatic inflammation by suppressing pro-inflammatory signaling along the gut-liver axis. An engineered probiotic provides further protection by chelating cadmium and enhancing antioxidant defenses, offering a promising therapeutic strategy (128, 129). Chronic cadmium exposure suppresses hepatic METTL3 expression and global m^6^A RNA methylation. Overexpression of METTL3 alleviates Cd-induced steatosis, fibrosis, and hepatic stellate cell activation. Transcriptomic analysis identifies key METTL3-regulated pathways disrupted by Cd, highlighting m^6^A-dependent epigenetic regulation as a central mechanism in cadmium-induced hepatotoxicity (130). Chronic cadmium exposure induces hepatic dysfunction and gut microbiota dysbiosis in mice. Selenium-enriched *Lactobacillus plantarum* (LS) reduces hepatic cadmium accumulation by 49.5% and more effectively attenuates oxidative stress and inflammation than either selenium nanoparticles or the native strain. LS demonstrates significant protection against Cd-induced hepatointestinal injury, supporting its potential as a therapeutic agent for heavy metal detoxification (131). Chronic cadmium exposure induces hepatotoxicity in chickens via oxidative stress and inflammation, which is effectively mitigated by green tea polyphenols. In aquatic systems, carbon quantum dots similarly cause gut-liver axis damage in fish. These findings reveal shared toxicity pathways across species and underscore the protective potential of natural polyphenols (132, 133).

Rice bran insoluble dietary fiber (RBIDF) alleviates cadmium toxicity by reducing intestinal cadmium absorption and enhancing its fecal excretion. It simultaneously restores gut microbiota balance, enriching beneficial bacteria such as *Lactobacillus* while suppressing harmful species. This dual mechanism establishes RBIDF as an effective dietary intervention against cadmium-induced damage (134). Morin, a natural flavonoid, protects rats against acute cadmium-induced hepatotoxicity by restoring liver function and reducing oxidative and endoplasmic reticulum stress in a dose-dependent manner. It modulates inflammatory and apoptotic markers, enhances antioxidant defenses, and suppresses JNK/p-PERK and Caspase-12 signaling. These findings demonstrate Morin’s therapeutic potential for treating heavy metal–induced liver injury (135). Low-dose cadmium exposure unexpectedly enhanced cattle growth by improving the feed-to-gain ratio. This hormetic effect was linked to increased rumen microbial activity and enrichment of short-chain fatty acid-producing bacteria such as *Prevotella*. These findings suggest cadmium can induce adaptive microbial restructuring that improves energy harvest (136). Chronic exposure to polystyrene microplastics (PS-MPs) and cadmium induces liver injury in mice, yet their combined exposure paradoxically reduces toxicity compared to cadmium alone. PS-MPs activate both protective (Nrf2-Keap1) and apoptotic (p53–Bcl-2/Bax) pathways. Separately, a zinc-quercetin complex demonstrates potent hepatoprotection against cadmium toxicity by alleviating oxidative stress and genotoxicity (137, 138). *Dendrobium officinale* polysaccharide (DOP) attenuates liver fibrosis by preserving intestinal barrier integrity and upregulating tight junction proteins. It subsequently suppresses the hepatic LPS–TLR4–NF-κB signaling pathway, reducing inflammation and fibrotic progression. These findings demonstrate DOP’s protective role through modulation of the gut-liver axis (139). Diet is a primary determinant of gut microbiota composition, which subsequently modulates inflammatory pathways in chronic diseases. Health-promoting dietary patterns support microbial balance and reduce systemic inflammation, whereas Western diets often induce dysbiosis. Targeting the diet-microbiota-barrier axis therefore represents a promising therapeutic strategy for chronic inflammatory disorders (140, 141). Kudzu-resistant starch alleviates NAFLD by enhancing gut barrier function and inhibiting LPS/TLR4-mediated inflammation. It further restores microbiota diversity and regulates lipid metabolism through key targets such as SREBP-1c. This multi-target mechanism positions it as a promising dietary intervention, in contrast to high-fat diets that exacerbate gut dysfunction (142, 143). Co-exposure to microplastics and tetracycline induces severe intestinal damage and inflammation in young mice, while concurrently enriching antibiotic resistance genes. This combination also disrupts human cell viability and gut microbial composition *in vitro*. These findings demonstrate how pollutant mixtures can synergistically amplify damage to both host and microbial function (144, 145). Cadmium induces multi-organ toxicity, primarily targeting the liver by disrupting hepatocyte function and activating cell death pathways. This disruption promotes fatty liver disease and hepatocellular carcinoma through interference with critical processes such as autophagy (**Table 3**). Understanding these mechanisms provides key insights into the pathology of cadmium-induced hepatotoxicity (158). High-dose intraperitoneal N-acetylcysteine (800 mg/kg) induced acute liver, kidney, and spleen damage in mice, leading to mortality. This dose increased oxidative stress, disrupted lipid metabolism, and suppressed ATP production. In contrast, a lower dose (275 mg/kg) enhanced antioxidant capacity and maintained metabolic balance. These findings highlight the dose-dependent toxicity of NAC and emphasize the need for cautious dosing, particularly in healthy individuals (159).

**Table 3:** Gut–liver axis disruption by cadmium induced. “↓” indicates downregulation (decrease). “↑” indicates upregulation (increase).

Exposure route	Gut-level disruption	Translocation mechanism	Liver injury trigger	Molecular pathway	Hepatotoxic consequence	Therapeutic target
Oral Ingestion	Damages tight junctions (ZO-1, occludin)	Paracellular leakage into portal circulation	Endotoxin (LPS) influx	TLR4/NF-κB	Kupffer cell activation; ↑ TNF-α, IL-6	Probiotics; Zinc supplementation (146).
Diet/Food	Reduces mucin-2 secretion	Increased gut permeability	Bacterial translocation (e.g., *E. coli*)	NLRP3 inflammasome	Pyroptosis; hepatic inflammation	Mucosal protectants (e.g., glutamine) (147).
Water/Soil	Alters gut microbiota diversity	Microbial dysbiosis → bile acid metabolism disruption	Toxic bile acid accumulation	FXR signaling impairment	Cholestasis; oxidative stress	FXR agonists (e.g., obeticholic acid) (148).
Chronic Low-Dose	Induces intestinal apoptosis	Damaged enterocytes → luminal cadmium absorption	Cd accumulation in hepatocytes	Metallothionein saturation	Necrosis; mitochondrial dysfunction	Chelation therapy (EDTA/DMSA) (149).
Acute High-Dose	Disrupts redox balance (↓ GSH)	Cd-bound metallothionein portal transport	ROS overproduction	Nrf2/Keap1 suppression	Lipid peroxidation; DNA damage	N-acetylcysteine (NAC) (150).
Enterohepatic Recirculation	Bile excretion of Cd	Cd reabsorption in ileum	Secondary liver exposure	BSEP transporter inhibition	Persistent hepatocyte damage	Cholestyramine (bile acid sequestrant) (151, 152).
Immune Crosstalk	Activates gut dendritic cells	Portal delivery of cytokines	Hepatic stellate cell (HSC) activation	TGF-β/Smad	Collagen deposition; fibrosis	Galectin-3 inhibitors (153).
Vascular Link	Intestinal oxidative stress	Portal endotoxemia	Sinusoidal endothelial cell death	eNOS downregulation	Microcirculation dysfunction	Antioxidants (selenium/vitamin E) (154).
Metabolic Dysfunction	Short-chain fatty acid (SCFA) reduction	↓ Butyrate → disrupted gut-liver signaling	Altered hepatic glycolysis	AMPK/PGC-1α inhibition	Steatosis; energy depletion	SCFA supplementation (155).
Zinc Competition	Cd binds zinc transporters (Zip4)	Zinc deficiency → gut barrier failure	Zn-Cd substitution in liver enzymes	MTF-1 dysregulation	Impaired detoxification capacity	Zinc-rich diets (156).
Systemic Inflammation	Gut-derived exosomes with Cd	Systemic circulation	Macrophage infiltration	CXCL1/CXCR2 axis	Lobular necrosis; ALT elevation	Anti-IL-1β antibodies (157).

Exposure to 250 MeV protons induces dose-dependent apoptosis in the murine small intestine, with notable hypersensitivity observed at doses as low as 0.1 Gy. Proton irradiation significantly reduces crypt viability, mucosal surface area, and cellular proliferation. Gene expression profiling revealed distinct apoptotic pathways: low-dose exposure triggers apoptosis through cellular stress responses, while high-dose exposure primarily activates it via direct DNA damage (160). Tumor Necrosis Factor (TNF) is a pivotal inflammatory mediator regulated by epigenetic mechanisms and microRNAs. Although its tumoricidal properties hold therapeutic promise, its clinical application in lower gastrointestinal diseases and cancers remains challenging. A detailed understanding of TNF signaling is essential for addressing these clinical obstacles (161). Long-term administration of enteric-coated aspirin in mice induced chronic small intestinal injury characterized by oxidative stress, barrier dysfunction, and inflammation. It disrupted mitochondrial integrity and impaired antioxidant defenses via the Nrf2/Gpx4 pathway. Aspirin also increased intestinal permeability and altered gut microbiota composition, notably depleting *Akkermansia* and *Lactobacillus*. This model clarifies key mechanisms underlying aspirin-associated enteropathy (162).

Metallothionein-3 (MT3), a brain-specific metalloprotein, serves as a primary antioxidant that rapidly responds to oxidative stress. Its oxidation behavior varies between domain-specific and global patterns depending on its metalation state, with Zn(II) conferring protective effects. Thermodynamic studies reveal that MT3 binds Cu^+^ through enthalpy-driven processes and Zn²^+^ via entropy-driven mechanisms, forming Cu_4_–thiolate clusters. These properties underpin MT3’s critical role in neuronal copper buffering and oxidative defense (150, 163). Liver fibrosis progression is governed by bidirectional crosstalk between T cells and hepatic stellate cells (HSCs). CD4^+^, CD8^+^, and γδ T cells drive inflammation and activate HSCs, which in turn regulate T cell differentiation and proliferation. This reciprocal interaction of the novel therapeutic targets for fibrosis treatment (164). Hepatic fibrosis, a dynamic and potentially reversible process, arises from chronic liver injury where immune cells and hepatic stellate cells drive excessive extracellular matrix deposition. Understanding these immune-fibrotic interactions reveals promising therapeutic targets for liver disease treatment (165, 166). Short-chain fatty acids (SCFAs) alleviate liver injury by enhancing gut barrier function and reducing intestinal permeability. Evidence from murine models demonstrates that SCFA supplementation consistently improves liver health, supporting the therapeutic potential of SCFA-based interventions for human liver disease and warranting clinical investigation (167, 168). Cadmium induces hepatotoxicity primarily by disrupting the gut-liver axis, compromising intestinal barrier function and triggering hepatic inflammation. Its toxicity involves key pathways like Nrf2 and FXR, and can be exacerbated or alleviated by co-exposure to other pollutants. Interventions using specific dietary components and engineered probiotics show promise in restoring gut-liver homeostasis and mitigating cadmium-induced damage.

## 6 Morin hydrate as an adjuvant to overcome cisplatin resistance and toxicity in liver cancer

**Figure 4:**
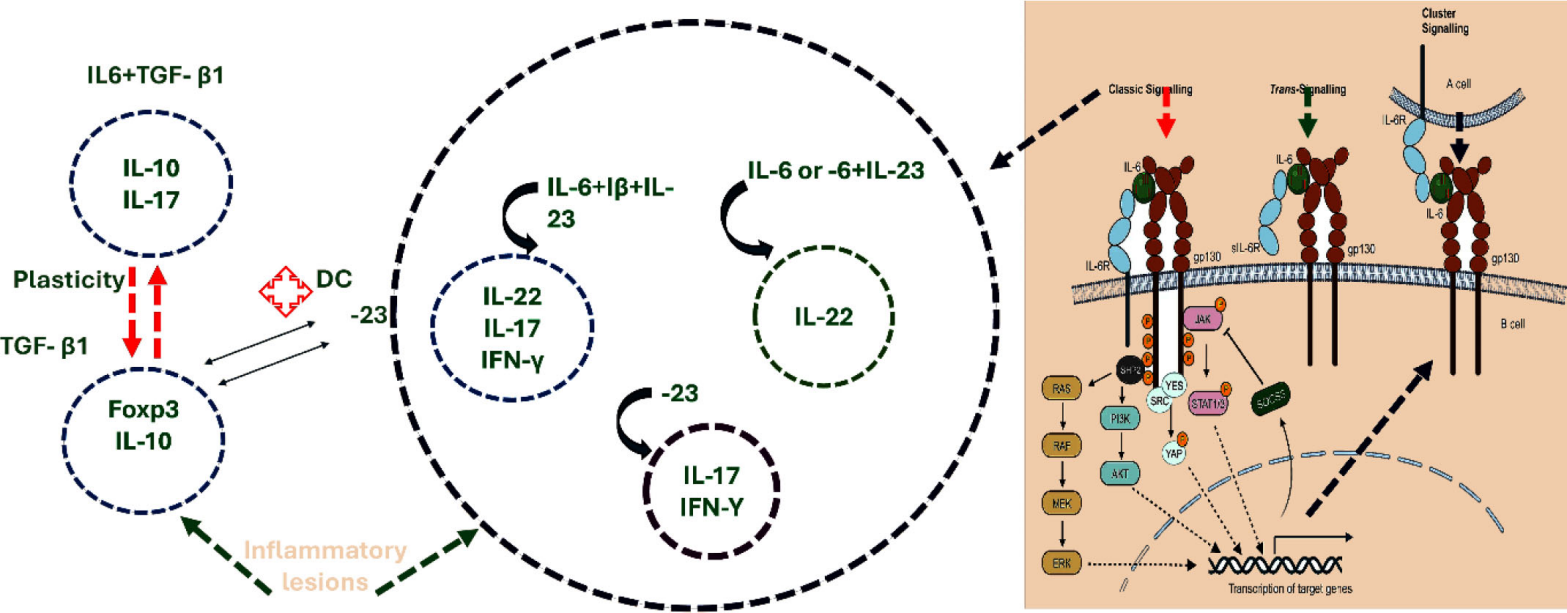
Signaling Diversity of IL-6 mediates immune regulation via classic (membrane IL-6R), trans-, and cluster signaling through IL-6R/gp130 complexes. Functional Outcomes of pathways dictate T cell plasticity and cytokine expression (e.g., IL-10, IL-17, IL-22, IFN-γ). Downstream Cascades. Signaling engages JAK/STAT, PI3K/AKT, and MAPK pathways, regulating immunity and inflammation. https://www.biorender.com/.

Cisplatin resistance in liver cancer is driven by PARP1-mediated autophagy via the HMGB1 pathway. Morin hydrate overcomes this resistance by simultaneously inhibiting PARP1 and blocking protective autophagy, thereby synergistically enhancing cisplatin’s cytotoxicity. This combination demonstrates significant tumor suppression, positioning morin hydrate as a promising adjuvant for resistant cancers (169). High-dose cisplatin induces significant renal and hepatic injury. Morin hydrate (100 mg/kg) protects against this renohepatic damage through antioxidant and anti-inflammatory mechanisms, restoring organ function, reducing oxidative stress, and preventing histological damage. These findings support morin hydrate’s potential as an adjuvant therapy to mitigate cisplatin-induced toxicity (170). The flavonoid morin modulates key pathways governing inflammation and cell survival, offering multi-target pharmacological effects. As cisplatin remains a standard treatment for advanced liver cancer, its efficacy is often limited by acquired resistance. This positions morin as a promising adjuvant to enhance cisplatin-based regimens for hepatobiliary cancers (171, 172). The natural flavonoid morin exhibits potent antioxidant and anti-inflammatory activities, demonstrating therapeutic potential against various pathologies including cancer and neurological injury. Its efficacy stems from the modulation of multiple signaling pathways related to oxidative stress and cell survival. Current evidence firmly supports morin as a promising candidate for further therapeutic development (173). A novel poly(2-oxazoline) micelle system enables efficient co-delivery of etoposide and platinum drugs with over 50% dual-drug loading. Its unique worm-like structure enhances drug release, pharmacokinetics, and tumor targeting. This system demonstrates significantly improved antitumor efficacy in both small cell and non-small cell lung cancer models (174). Hesperidin protects against ifosfamide-induced nephrotoxicity through its potent antioxidant and antiapoptotic properties. In a separate apoptotic pathway, TNF-induced cell death involves early caspase-9 activation that occurs independently of cytochrome c. This distinct mechanism amplifies the apoptotic cascade, even when protective proteins like Bcl-xL are present (175, 176). Apoptotic pathways including mitochondrial, death receptor, and ER-mediated mechanisms contribute to postmortem beef tenderization. Mitoquinone inhibits these signals, preserving muscle integrity and reducing tenderization. In contrast, the triazole-based compound IIIM(S)-RS98 induces apoptosis in lung cancer cells via mitochondrial and PI3K/Akt pathways (**Figure 4**).

**Figure 5:**
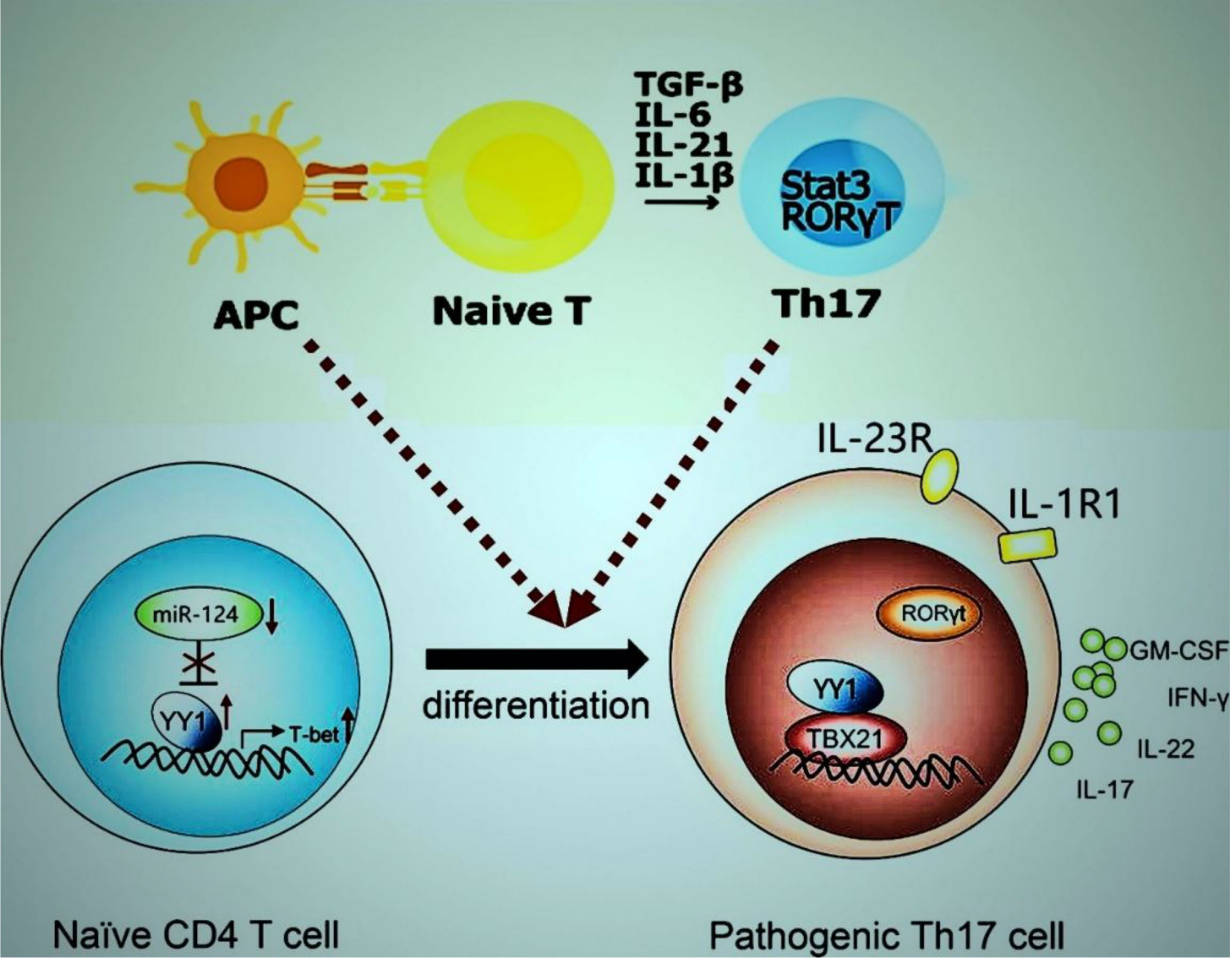
TGF-β, IL-6, IL-21, and IL-1β drive naïve CD4^+^ T cell differentiation into pathogenic Th17 cells via Stat3/RORγt activation, modulated by miR-124, YY1, and TBX21. Pathogenic Th17 cells produce proinflammatory cytokines. https://www.biorender.com/.

These findings demonstrate that mitoquinone and IIIM(S)-RS98 exert distinct, context-dependent regulatory effects on apoptosis in food science and oncology (177, 178). *Porphyromonas gingivalis* promotes atherosclerosis by inducing endothelial apoptosis. The synthetic RNA analog Poly(I:C) counteracts this effect by modulating Bcl-2 and caspase proteins, thereby protecting endothelial cells. This anti-apoptotic mechanism is mediated through JAK/STAT signaling pathway activation (179). Apaf-1 functions not only in apoptosis but also as an evolutionarily conserved cytosolic DNA sensor. Upon binding cytoplasmic DNA, it recruits RIP2 via its WD40 domain, activating NF-κB-mediated inflammation. This dual role enables Apaf-1 to coordinate the switch between apoptosis and inflammatory signaling, establishing it as a critical cell fate checkpoint (180). Cisplatin-induced exosomes selectively trigger caspase-9-dependent apoptosis in gastric cancer cells, revealing a targeted therapeutic mechanism. In contrast, chronic co-exposure to arsenic and chromium causes additive nephrotoxicity in zebrafish through oxidative stress and impaired DNA repair. These findings demonstrate both a novel cancer treatment strategy and the ecological risk of heavy metal mixtures (181, 182). In hepatocellular carcinoma, glutathione S-transferase Pi (GSTP) translocates to the nucleus and modulates redox signaling via the Nrf2-Keap1 complex. This interaction stabilizes Nrf2, promoting an antioxidant response that supports tumor survival. Consequently, GSTP inhibition effectively induces apoptosis, revealing its role as a key regulatory node in liver cancer (183). Myoglobin triggers ferroptosis in renal tubular epithelial cells, contributing to Crush Syndrome-associated acute kidney injury. This process is characterized by elevated ACSL4 and reduced GPX4 levels, confirming ferroptotic activation. These findings establish ferroptosis as a key mechanism in myoglobin-induced nephrotoxicity (**Figure 5**).

Deferoxamine chelates iron and inhibits ACSL4, while rosmarinic acid activates Nrf2 by disrupting its Keap1 interaction. Their combined treatment synergistically activates the Nrf2/Keap1 pathway, effectively blocking myoglobin-induced ferroptosis. This dual approach demonstrates significant therapeutic efficacy against Crush Syndrome-associated acute kidney injury (184). Resistance to platinum-based chemotherapy in ovarian cancer frequently involves restored DNA repair capacity. PARP-1 contributes significantly by selectively binding to cisplatin-induced DNA damage, particularly 1, 2-d(GpG) crosslinks (**Table 4**). These findings reveal actionable targets for overcoming platinum resistance in ovarian cancer treatment (207).

**Table 4:** Cisplatin–morin synergistic mechanisms in hepatocellular carcinoma “↓” indicates downregulation (decrease). “↑” indicates upregulation (increase)..

Therapeutic mechanism	Cisplatin action	Morin hydrate action	Synergy mode	Primary target	Pathway modulation	Experimental model	Efficacy outcome	Toxicity reduction	Resistance impact	Clinical translation
Apoptosis Induction	DNA crosslinking → caspase-9 activation	↑ Bax/Bcl-2 ratio; ↓ survivin	Mitochondrial priming	Caspase-3/9	p53/PUMA axis	HepG2 xenografts	↑ Tumor shrinkage (82% vs. 48% monotherapy)	↓ Renal caspase-3 cleavage	Reverses cisplatin evasion	Enhanced tumor kill with lower nephrotoxicity (185).
ROS Management	Generates superoxide radicals	Scavenges OH⁻/O_2_⁻; ↑ SOD/Catalase	Redox balance restoration	Nrf2/KEAP1	ARE antioxidant response	Huh-7 cells + cisplatin	↑ Cancer cell death (ROS threshold)	↓ Hepatic lipid peroxidation	Counters antioxidant adaptation	Permits higher cisplatin doses safely (186).
DNA Repair Inhibition	Platinum-DNA adduct formation	PARP-1 suppression	Repair blockade	ERCC1-XPF complex	BER pathway disruption	HCC patient-derived organoids	↑ DNA fragmentation (2.3-fold)	↓ Genomic instability in non-target cells	Overcomes NER proficiency	Sensitizes repair-competent tumors (187).
Anti-Metastasis	Weak anti-migration effect	↓ MMP-9/2; ↑ TIMP-1	Invasion barrier	FAK/Src kinases	EMT reversal (↑ E-cadherin)	Lung metastasis model (MHCC97H)	↓ Micro-metastases (73% reduction)	Preserves endothelial integrity	Blocks adaptive motility	Adjuvant for advanced HCC (188).
Cell Cycle Arrest	G2/M phase arrest	CDK4/6 inhibition → G1/S block	Multi-phase arrest	Cyclin D1/p21	Rb pathway activation	PLC/PRF/5 cells	↑ G0/G1 (Morin) + G2/M (Cis) accumulation	Spares hematopoietic stem cells	Prevents checkpoint adaptation	Targets heterogeneous cell populations (189).
Autophagy Modulation	Induces protective autophagy	Inhibits autophagic flux (↓ LC3-II)	Blocks tumor survival mechanism	Beclin-1/ATG5	ULK1 complex regulation	Starvation-induced HCC cells	Switches autophagy → apoptosis	↓ Autophagic nephropathy	Collapses resistance mechanism	Overcomes hypoxia-induced resistance (190, 191).
Inflammation Control	↑ IL-6/IL-8 (pro-tumorigenic)	↓ NF-κB p65 nuclear translocation	Cytokine storm suppression	IκBα	JAK/STAT inhibition	DEN-induced HCC rats	↓ Serum TNF-α (68%)	↓ Kupffer cell activation	Reverses TME immunosuppression	Mitigates cachexia in late-stage HCC (192).
Angiogenesis Suppression	Weak VEGF inhibition	↓ HIF-1α; ↓ VEGFR2 phosphorylation	Dual angiogenic blockade	VEGF-A/PIGF	PI3K-Akt-eNOS axis	Chick chorioallantoic assay	↓ Microvessel density (81% vs. controls)	Maintains sinusoidal perfusion	Prevents revascularization	Inhibits post-resection recurrence (193).
Drug Efflux Reversal	Substrate of P-gp/ABCB1	P-glycoprotein downregulation	Efflux pump inhibition	ABCB1 transporter	MDR1 gene suppression	Doxorubicin-resistant Hep3B	↑ Cisplatin accumulation (3.1-fold)	Reduces biliary cisplatin excretion	Reverses transporter-mediated resistance	Resensitizes MDR tumors (194).
Mitochondrial Protection	Damages complex I → ATP depletion	↑ Cardiolipin stabilization	Selective cancer cell targeting	VDAC/ANT pores	MPTP closure regulation	Isolated liver mitochondria	↑ Cancer cell ATP crash (synergistic)	Preserves hepatocyte respiration	Exploits metabolic vulnerability	Reduces cisplatin-induced fatigue (195).
Ferroptosis Sensitization	Indirect iron accumulation	↑ ALOX15; ↓ GPX4	Lipid peroxidation boost	System xc⁻ transporter	Glutathione depletion	Erastin-treated Hepa1-6	↑ Ferroptotic death (additive to apoptosis)	Spares hepatic GPX4 reserves	Bypasses apoptotic resistance	Targets therapy-resistant stem cells (196).
Epigenetic Regulation	Histone H3 acetylation	HDAC2/8 inhibition	Chromatin remodeling	DNMT1/HDAC complexes	miRNA-34a restoration	HCC tissue microarray analysis	↓ DNMT1 (hypermethylation reversal)	Minimal global methylation changes	Re-silences oncogenes	Demethylates tumor suppressor genes (197).
MAPK Pathway Modulation	JNK/p38 activation (mixed effects)	ERK1/2 suppression	Stress signaling redirection	ASK1-Trx complex	Balanced JNK/ERK crosstalk	Sorafenib-resistant models	↑ Sustained JNK activation (pro-death)	↓ ERK-driven hepatocyte proliferation	Counters compensatory survival	Prevents adaptive MAPK rewiring (198).
Glucose Metabolism	↑ Warburg effect (compensatory)	↓ HK2/GLUT1; ↑ PDK4	Metabolic inflexibility	Hexokinase-II	HIF-1α/LDHA axis	FDG-PET in orthotopic tumors	↓ Glycolytic flux (PET SUV_max_ 52%↓)	Maintains normal hepatocyte glucose use	Starves hypoxic niches	Enhances radiation sensitivity (199).
Cellular Senescence	Induces senescence (therapy escape)	↓ p16INK4a; ↑ BUBR1	Senescence bypass	p53/p21 axis	SASP factor inhibition	Aged HCC microenvironment	Converts senescence → apoptosis	↓ Senescent hepatocyte accumulation	Prevents paracrine resistance	Reduces recurrence in elderly patients (200).
Immunogenic Cell Death	Calreticulin exposure	↑ HMGB1 release	Enhanced DC maturation	TLR4/CD91 receptors	STING pathway activation	Syngeneic mouse HCC (H22)	↑ CD8^+^ T-cell infiltration (4-fold)	↓ T-reg recruitment	Reverses immune desert	Complements checkpoint inhibitors (201).
Bile Acid Homeostasis	Disrupts bile acid transporters	↑ FXR/SHP; ↓ CYP7A1	Hepatobiliary protection	NTCP/BSEP pumps	FGF19 signaling	Cholestatic hepatotoxicity model	Maintained bile flow (synergy group)	↓ Cisplatin-induced cholestasis	Prevents adaptive BA dysregulation	Enables longer treatment cycles (202).
Tissue Regeneration	Inhibits hepatocyte proliferation	↑ HGF/MET; ↓ TGF-β1	Compensatory repair	EGFR/STAT3	IL-6/gp130 regulation	Partial hepatectomy model	Accelerated liver recovery (proliferation ↑35%)	↓ Fibrosis progression	Balances kill/repair	Supports surgical resection combos (203, 204)
Pharmacokinetics	Rapid renal clearance	↑ Organic anion uptake (OATPs)	Hepatic concentration boost	OATP1B3 transporter	Albumin binding modulation	Perfused rat liver	↑ Liver cisplatin AUC (157%)	↓ Systemic C_max_ (29%)	Optimizes tumor exposure	Allows reduced systemic dosing (205, 206).

Blocking the IL-10 receptor enhances chemotherapy efficacy by restoring IL-12-mediated antitumor immunity. The potency of mesothelin-targeted T cells is further amplified by regulatory T cell depletion. These immunotherapeutic strategies demonstrate significant efficacy in the Panc02 pancreatic cancer model (208, 209). NTCP and BSEP, key bile acid transporters, show therapeutic relevance in liver diseases. Following liver resection, elevated bile salts activate FXR and upregulate IL-6/STAT3/c-JUN signaling to promote regeneration. In disparity, FGF19 remains unchanged, indicating its limited role in early human liver regeneration (210, 211).

Liver regeneration is coordinated by hepatocyte proliferation and regulated cell death through the IL-6/JAK/STAT3 and PI3K/PDK1/Akt pathways. These cascades function cooperatively to promote hepatocyte survival and replication during regeneration. However, the precise regulatory mechanisms of cell death in this process require further investigation (212). Hepatocyte growth factor promotes cardiac repair after myocardial infarction via c-Met signaling, demonstrating therapeutic potential for heart disease. In acetaminophen-induced acute liver failure, the capacity for hepatic regeneration critically determines survival. Targeting these regenerative pathways offers a promising strategy for treating both cardiac and hepatic injury (213, 214). Hepatic stellate cells (HSCs) are central drivers of liver fibrosis and cancer progression, differentiating into myofibroblasts and cancer-associated fibroblasts. Therapeutically targeting HSCs through surface markers enables precise drug delivery to these pathogenic cells. Rapidly evolving omics technologies continue to uncover novel HSC-specific targets for liver disease treatment (215). The tumor-specific isoform ct-OATP1B3-V1 enhances chemotherapy sensitivity by localizing to the plasma membrane and importing drugs. However, it simultaneously confers a proliferative advantage to cancer cells, creating a therapeutic paradox. This dual functionality establishes it as a complex yet promising target for cancer treatment (216). Organic anion transporting polypeptides (OATPs) critically mediate hepatic statin uptake, with transport efficiency varying by OATP subtype and statin properties. OATP1B1 exhibits the broadest substrate specificity, while other subtypes display more selective affinities. These insights help refine pharmacokinetic models and improve personalized statin dosing predictions (205, 217). Cisplatin’s efficacy in liver cancer is limited by resistance and toxicity, but morin hydrate acts as a potent adjuvant to overcome these barriers. It sensitizes tumors by inhibiting PARP1-mediated autophagy while reducing cisplatin-induced organ damage. This dual action synergistically enhances tumor cell death and improves the therapeutic index.

## 7 Limitations and future prospects

The clinical translation of morin and similar phytochemicals faces significant challenges, including poor bioavailability, limited human pharmacokinetic data, and natural product variability. Current reliance on preclinical evidence underscores the urgent need for rigorous clinical trials. Future progress requires developing advanced delivery systems, applying multi-omics approaches to elucidate mechanisms, investigating synergistic drug combinations, and exploring gut microbiota interactions. Realizing the potential of these multi-target agents will depend on sustained interdisciplinary collaboration to bridge laboratory findings and clinical applications. The molecular gut–liver signaling, we substantially revised the manuscript with detailed discussions of key pathways: TLR4/NF-κB, MAPK signaling, tight junction regulation, microbial metabolite signaling (e.g., SCFAs), and cytokine cascades. These additions strengthen mechanistic links between intestinal dysfunction and hepatic pathology. Regarding avian/fish model limitations, we incorporated human clinical data on biomarkers, microbiome profiling, and liver disease outcomes to enhance translational relevance.

### 7.1 Identification of gut–liver axis–related therapeutic targets

The future studies to systematically identify key molecular targets involved in gut–liver communication during cadmium exposure, including intestinal barrier regulators, bile acid signaling pathways, microbial metabolites, and inflammatory mediators, which may serve as potential intervention targets.

### 7.2 Integration of multi-omics approaches

We proposed that future research should combine metagenomics, metabolomics, and transcriptomics to better elucidate the complex interactions between gut microbiota alterations and hepatic metabolic dysfunction in cadmium-induced liver injury.

### 7.3 Development of combined intervention strategies

The revised section now discusses the potential of combined therapeutic approaches, such as the co-administration of natural bioactive compounds with probiotics or prebiotics, to synergistically regulate gut microbiota composition, restore intestinal barrier integrity, and reduce hepatic oxidative stress and inflammation.

### 7.4 Improvement of translational and clinical research

We also emphasize the importance of well-designed animal studies and clinical investigations to validate the safety, efficacy, and mechanisms of gut–liver axis–targeted interventions, thereby facilitating their potential translation into clinical applications.

## 8 Clinical implications and conclusion

The increasing global burden of liver disease necessitates effective, multi-targeted therapies. Natural compounds such as morin are promising candidates, capable of simultaneously modulating oxidative stress, inflammation, fibrosis, and apoptosis. A key therapeutic advantage lies in their dual capacity to sensitize tumors to chemotherapy while protecting healthy tissues from damage. However, widespread clinical adoption is hindered by challenges including low bioavailability and insufficient clinical trial data. Realizing their full potential requires an interdisciplinary strategy to overcome these translational barriers and integrate phytochemicals into modern treatment paradigms.
